# Motivations and Experiences of Older Adult Volunteers in a Telehealth Nursing Simulation Activity

**DOI:** 10.1093/geroni/igab046.3415

**Published:** 2021-12-17

**Authors:** Jennifer Crittenden, Kayla Thompson

**Affiliations:** 1 University of Maine, University of Maine, Maine, United States; 2 University of Maine, Bangor, Maine, United States

## Abstract

The COVID-19 pandemic has posed challenges to safely engaging older adults in volunteer activities. This research explored a unique partnership between a Retired Senior and Volunteer Program (RSVP) and a school of nursing to administer a telehealth virtual simulation training for nurse practitioner students. Semi-structured interviews were carried out with nursing simulation coordinators and volunteers after the telehealth simulation exercise. The purpose of this research was to identify principles of successful virtual volunteer engagement for telehealth simulations. This initial pilot study encompassed debriefing interviews with volunteers (N = 3) and interviews with simulation coordinators (N = 2). Three major themes emerged within the response coding: 1) the benefits of virtual simulation volunteering, 2) technology as a facilitating factor and challenge, and 3) unique volunteer management considerations. Both volunteers and coordinators noted that volunteers derived positive emotional benefits and new insights from their participation. Coordinators discussed the “authenticity” factor that older adults brought to the simulation experience as a benefit to engaging older adult volunteers. Technology sub-themes included accessibility considerations, experience with the online format, and other logistical considerations in conducting telehealth simulation. Volunteer management sub-themes encompassed volunteer skills and motivations, the perceived successful aspects of training, and improvements for future simulations. Volunteers discussed an interest and connection to healthcare and education as a motivating factor for their participation in the telehealth simulation. This small scale pilot research will be expanded through future simulation activities to continue to identify principles of practice for engaging older adults in virtual volunteerism.

